# The use of transgenic animals for xenotransplantation: An update

**DOI:** 10.1002/ame2.70105

**Published:** 2025-11-27

**Authors:** Julia Motławska, Ana Amaral, Marta Cerveira‐Pinto, Paweł Kordowitzki

**Affiliations:** ^1^ Department of Basic and Preclinical Sciences Faculty of Biological and Veterinary Sciences Nicolaus Copernicus University Torun Poland; ^2^ Faculty of Veterinary Medicine, CIISA—Centre for Interdisciplinary Research in Animal Health University of Lisbon Lisbon Portugal; ^3^ Associate Laboratory for Animal and Veterinary Sciences (AL4AnimalS) Lisbon Portugal

**Keywords:** editing modifications, organ shortage, pigs, transgenic animals, xenotransplantation

## Abstract

Xenotransplantation, that is, the transplantation of cells, tissues, and organs between species, is a rapidly developing alternative to classical transplantology in human medicine. Since the first successful kidney transplant in 1954, transplant medicine has made enormous progress. Until today, there are numerous patients worldwide waiting for an organ to be transplanted, and the number is still increasing, whereas the number of available organs is decreasing. One promising solution to this critical issue is the breeding of genetically modified animals as potential donors, which has gained the attention of scientists over the past two decades. Recent advancements in xenotransplantation have led to successful transfers of genetically modified pig organs into human recipients. Particularly, pig kidneys have been transplanted into living humans, demonstrating normal postsurgical function. Additionally, pig lungs functioned for 9 days in a brain‐dead individual without experiencing hyperacute rejection. Furthermore, the successful xenotransplantation of pig hearts into living persons, exhibiting life‐sustaining graft function, underscores significant progress toward clinically viable xenotransplants. This review provides an updated overview of the animal species and models used in xenotransplantation, with particular emphasis on the potential of transgenic pigs as donors. It discusses the process involved in producing the aforementioned animals, including the methods used to modify their genome. Particular attention is paid to immunological and genetic barriers, as well as zoonotic risks, and the possibilities and limitations of this technology. Although xenotransplantation is still in its experimental stage, it may play a crucial role in saving patients' lives in the future.

## Graphical abstract

One way to produce genetically modified pigs is to use somatic cell nuclear transfer (SCNT). This procedure involves removing the metaphase plate from a mature oocyte using micromanipulation and then transferring the modified nucleus from the donor somatic cell to the oocyte. It is then artificially activated, either chemically or electrically, triggering further embryo development. Such embryos are transferred to the fallopian tubes or cultured to the blastocyst stage and transferred to the uterus. Cloned embryos have a lower implantation rate compared to embryos resulting from fertilization, which translates into increased costs of the procedure. This phenomenon can be partially compensated for by transferring a larger number of embryos, as pigs naturally compensate for excess through the physiological resorption of supernumerary embryos. Another limitation of this technique is that pregnancies obtained using SCNT embryos are often prolonged and may require induction of labor or cesarean section. Furthermore, their offspring tend to be more vulnerable, requiring special care and attention to reduce the significantly higher rates of stillbirth and neonatal mortality.

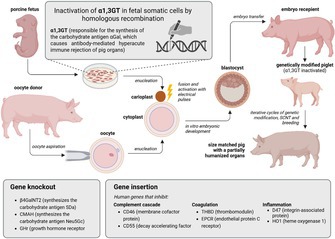

## 1 INTRODUCTION

The development of transplantology began with a groundbreaking kidney transplant in 1954, when rejection of the organ was avoided due to the identical genetic material of identical twins.^1^ This success motivated scientists to investigate immunosuppression methods and to understand the immunological processes that occur in the body after transplantation. After the initial successes in this field, intensive work began on transplanting other organs, such as cardiac tissues, the heart, or the liver.^2^, ^3^, ^4^ Over the years, knowledge of the biological processes accompanying transplants has increased, and immunosuppression strategies have been refined to significantly reduce the risk of organ rejection.

Despite enormous progress in this field, transplantology still faces a significant problem: a shortage of organs for transplantation. According to official data, in the United States, more than 103 000 patients are on the waiting list for a transplantation, and more than 48 000 transplants were performed in 2024.^5^ According to data from Scandiatransplant, at the end of 2024, 2360 patients were waiting for a transplant in the Scandinavian countries (Denmark, Finland, Iceland, Norway, Sweden, and Estonia), and 2153 underwent transplantation.^6^ Other European countries, such as Germany, the Netherlands, and Slovenia, have established a joint organization called Eurotransplant. According to data from this network, in 2024, over 13 572 patients were waiting for a transplant, yet only about 6200 received one.^7^

**FIGURE 1:**
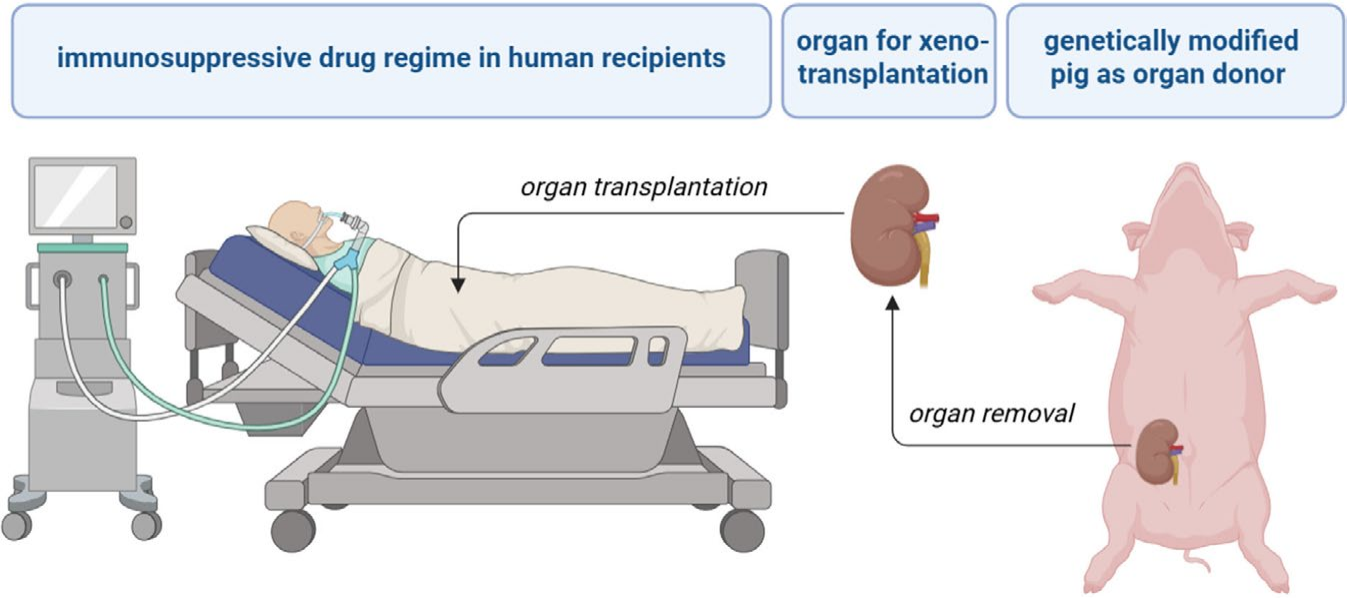
Scheme showing the main procedure of xenotransplantation.

Other European countries have independent national transplant systems. To increase the number of available organs, investigations into interspecies transplants, known as xenotransplantation (Figure 1), have been conducted. Therefore, transgenic animals are genetically modified by silencing specific genes or introducing selected human genes necessary to obtain organs that are more immunologically compatible with the recipient's body.^8^ This review comprehensively examines the animal species and models employed in xenotransplantation, highlighting the promising role of transgenic pigs as potential organ donors. Furthermore, it delves into the methodologies involved in producing these genetically modified animals, specifically detailing the techniques for genomic alteration. Significant consideration is given to overcoming immunological and genetic incompatibilities, addressing potential zoonotic transmission risks, and exploring both the feasibility and inherent constraints of this therapeutic approach. Additionally, the ethical implications associated with xenotransplantation are thoroughly considered. Despite xenotransplantation remaining largely experimental, its future application holds substantial promise for improved availability of organs.

## 2 XENOTRANSPLANTATION

### 2.1 Classification of transplants

**FIGURE 2:**
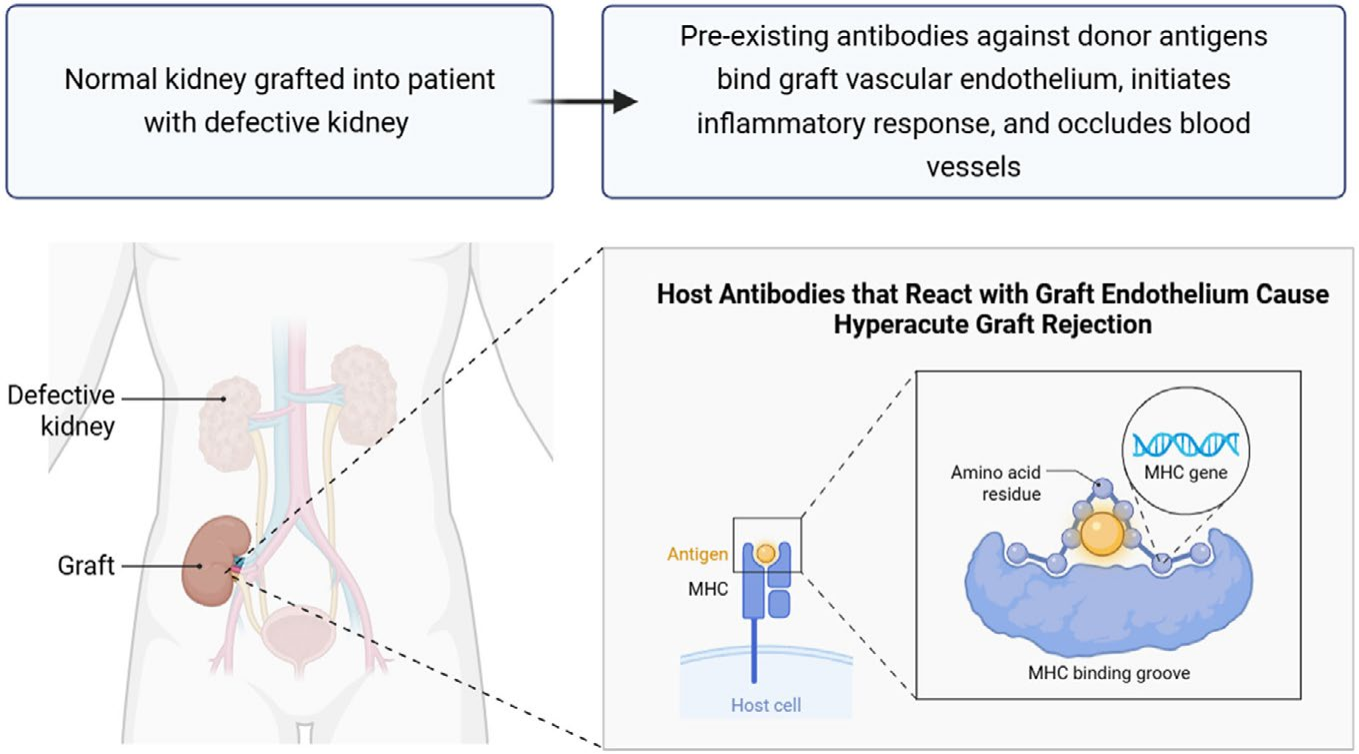
Scheme showing the process of major histocompatibility complex (MHC) activation upon xenograft recognition.

Genetic variances among the donor (animal) and recipient (human), especially in terms of major histocompatibility complex molecules (Figure 2), can trigger an immune response leading to transplant rejection. Therefore, the type of transplant, determined by the degree of genetic compatibility, is crucial to the success of the transplant. There are four main types of transplants: autogenous (autologous), where the donor and recipient are the same organism; isogenic (syngeneic), performed between genetically identical individuals, for example, monozygotic twins; allogeneic, referring to the transplantation among individuals of the same species but genetically different; and xenogeneic, referring to the transplantation among individuals of different species.^9^, ^10^

### 2.2 Animals used in xenotransplantation

Over the past few decades, numerous experiments have been conducted to identify the most suitable animal species for xenotransplantation. Primates are the first species considered for xenotransplantation. However, they are not used as organ donors for humans for many important reasons. The close relationship between primates and humans increases the risk of transmitting zoonotic diseases. Furthermore, their use raises significant ethical concerns and is subject to legal restrictions in many countries. Furthermore, primates have a longer reproductive cycle and higher breeding and care requirements than pigs, for example.^11^, ^12^ Although primates are not used as organ donors, baboons and macaques play an important role as preclinical models. In studies of this type, pig organs are transplanted into them, which allows the recipient's immune response, transplant function, and possible complications to be assessed before clinical trials involving humans begin.^13^, ^14^

Numerous studies have established that pigs are optimal organ donors due to several advantageous characteristics, including ease of breeding, the ability to produce numerous litters, and organs that are anatomically and physiologically very similar to those of humans. In addition, as pigs are commonly used in the food industry, their use in medicine raises relatively fewer ethical controversies.^15^ However, before pig organs can be used clinically, several major obstacles must be overcome, including immunological rejection reactions, the transmission of zoonotic pathogens, and physiological differences between pig and human organs.^16^ Bovine pericardium is widely used as a xenograft material for heart valves, particularly in surgical and transcatheter bioprosthetic valves. Its favorable mechanical properties, hemodynamic performance, and relative ease of processing make it the standard choice for heart valves.^17^

### 2.3 Immunological consequences

The primary obstacle to xenotransplantation is the strong immunological barrier resulting from interspecies incompatibility, which leads to transplant rejection.^18^, ^19^ The most important problems include:

- Hyperacute rejection (HAR) is caused by the presence of preformed antibodies against α1,3‐galactose and other antigens such as Gal (Neu5Gc), leading to complement cascade activation.
- Acute humoral xenograft rejection results from de novo formation of antibodies against α1,3‐galactose and antigens other than Gal (Neu5Gc), leading to complement cascade activation. Endothelial cell activation, thrombotic microangiopathy, and coagulopathy all play a crucial role in this process.
- Immune cell‐mediated rejection involves the activation of natural killer cells and T lymphocytes.
- Immediate blood‐mediated inflammatory response is the result of a reaction to surface proteins, mediated by complement, innate immunity, platelet activation, and leukocytes.^20^

Important elements of immune response in the context of transplant rejection include the presence of natural antibodies that recognize structures on cells of the endothelium in transplanted organs, leading to complement activation, and the lack of appropriate complement inhibitors in donor cells, which increases their susceptibility to the cytotoxic effects of this system.^18^, ^19^, ^20^ The natural antibodies present in humans, responsible for HAR of pig transplants, are most often directed against antigens containing the disaccharide galactose‐α1,3‐galactose (Galα1‐3Gal), known as the Gal epitope. Its formation depends on the activity of the enzyme α1,3‐galactosyltransferase, which is not present in humans and apes, meaning these sugar residues are not produced in them.^18^, ^19^

In addition, pig cells express other carbohydrate antigens, such as *N*‐glycolylneuraminic acid (Neu5Gc) and the Sda, against which natural antibodies exist in humans and some nonhuman primates (NHP). The binding of these antibodies to target antigens after transplantation initiates complement activation, endothelial cell activation, clotting processes, and the so‐called antibody‐dependent cellular cytotoxicity.^19^, ^21^

### 2.4 Somatic cell nuclear transfer and genetic engineering of pigs

To inhibit rejection and prolong the survival of organs transplanted from pigs, xenoreactive antibodies are removed from the circulation, and transgenic animals lacking the major carbohydrate antigens and expressing one or more human complement pathway proteins are produced. It has been proven that pigs lacking the genes responsible for α‐Gal, Neu5Gc, and Sda exhibited reduced binding of human antibodies in vitro.^22^ Particularly, when organs from wild‐type (nongenetically modified) pigs were transplanted into primates, and even more so into humans, a rapid rejection, referred to as HAR, occurred.^18^, ^19^

A key challenge remains the constant growth of the porcine organs after transplantation, which is especially relevant for the heart. Using genome editing, the knockout (k.o.) of the growth hormone receptor in pigs has been shown to reduce the respective size; however, this approach often results in poor health and low longevity.^23^ A more promising solution is to use naturally smaller pig breeds or genetically modified minipigs.^24^, ^25^ Thus, miniature pigs are considered the best candidates for organ donors because their organ sizes are similar to those of humans. Furthermore, some studies suggest that miniature pigs are better embryo recipients than domestic pigs in cloning procedures.^26^ In addition, miniature pigs are preferred because they are easy to handle, consume less feed, and require less space.^27^ The most commonly used breeds are Gottingen, Sinclair, Yucatan, and Hanford.^28^

**FIGURE 3:**
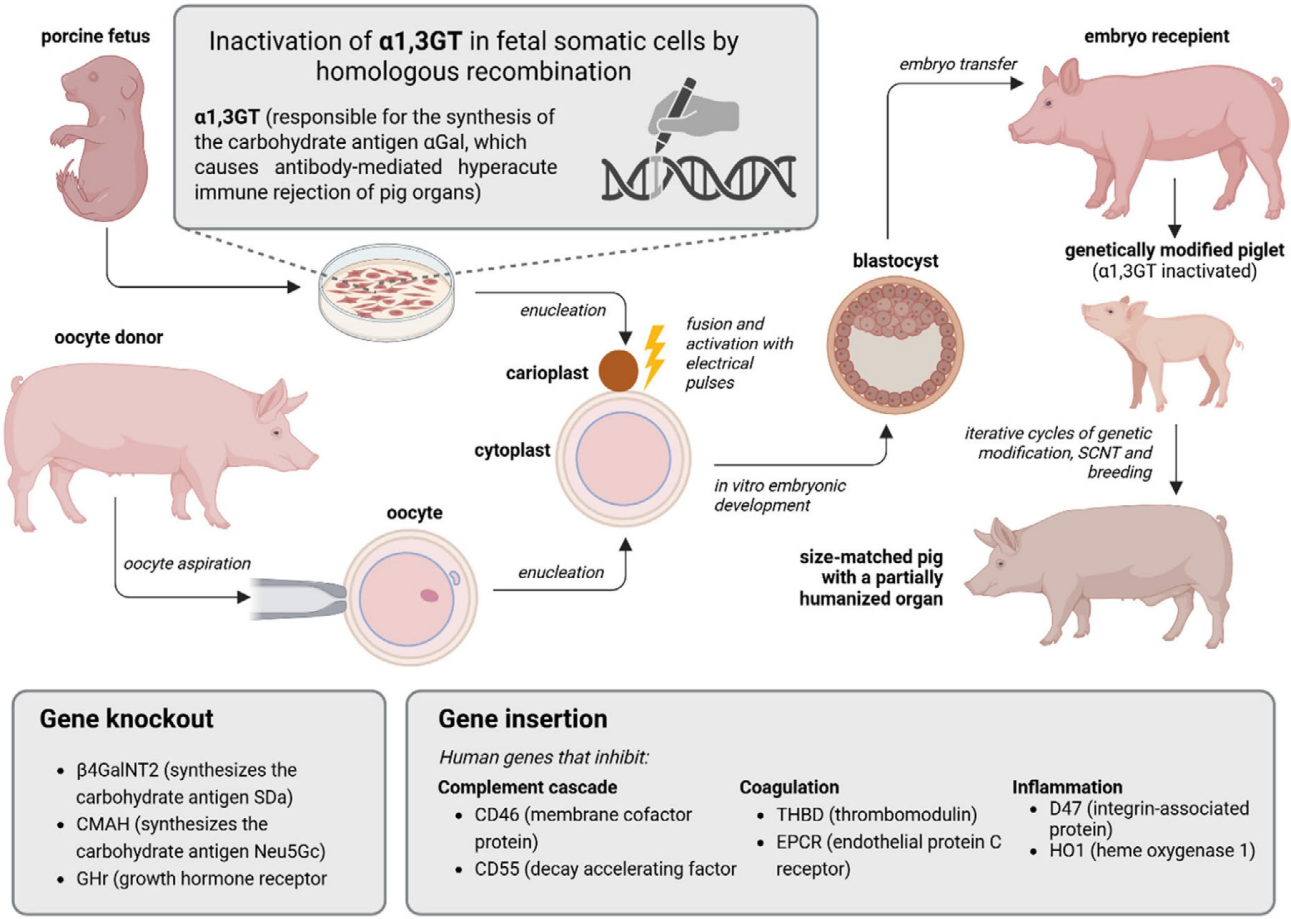
Scheme showing the main steps of somatic cell nuclear transfer (SCNT) and the production of genetically modified pigs for xenotransplantation. Adapted from Peterson et al.^32^

Among other techniques, genetically modified pigs can be produced using somatic cell nuclear transfer (SCNT) (Figure 3).^20^ This procedure involves removing the metaphase plate from a mature oocyte using micromanipulation and then transferring the modified nucleus from the donor somatic cell to the oocyte. It is then artificially activated, either chemically or electrically, triggering further embryo development.^29^, ^30^ Such embryos are transferred to the fallopian tubes or cultured to the blastocyst stage and transferred to the uterus. Cloned embryos have a lower implantation rate compared to embryos resulting from fertilization, which translates into increased costs of the procedure. This phenomenon can be partially compensated for by transferring a larger number of preimplantation embryos, as pigs naturally compensate for excess through the physiological resorption of supernumerary embryos. Another limitation of this technique is that pregnancies due to SCNT embryos are often prolonged and may require induction of labor or cesarean section.^31^ Furthermore, their offspring tend to be more vulnerable, requiring special care and attention to reduce the significantly higher rates of stillbirth and neonatal mortality.^20^

Although protocols for oocyte in vitro maturation and embryo in vitro culture are well established, micromanipulation techniques remain technically demanding and labor intensive. In SCNT embryos, developmental competence is often reduced due to incomplete nuclear reprogramming, a biological process that is still not fully understood in livestock species.^33^ However, treatments that enhance chromatin remodeling, such as trichostatin A, have improved cloning efficiency in mice and show promising results in pigs.^34^, ^35^

Selecting appropriate donor cell lines has a significant impact on the efficiency of SCNT in the context of xenotransplantation. The ideal cell lines must possess genomic stability, high cloning efficiency, and the capacity to support normal embryonic development after transfer. Fetal fibroblasts (Figure 3) and adult somatic cells derived from genetically engineered pigs are commonly used, as they are accessible and highly responsive to genome‐editing techniques. This enables the incorporation of human‐compatible genetic modifications and the removal of immunogenic elements.^36^, ^37^ Moreover, optimizing in vitro culture conditions and thoroughly assessing genomic integrity help improve donor cell quality, ultimately enhancing the chances of producing viable and immunologically suitable xenografts via SCNT.^38^

#### 2.4.1 Modification of the somatic cell genome

In recent years, many different approaches to modifying the somatic cell genome have been developed. These modifications include the elimination or modification of genes responsible for the synthesis of molecules that trigger the major immune response, as well as the introduction of human genes, thereby improving the compatibility of porcine organs with the human immune system.^31^

**FIGURE 4:**
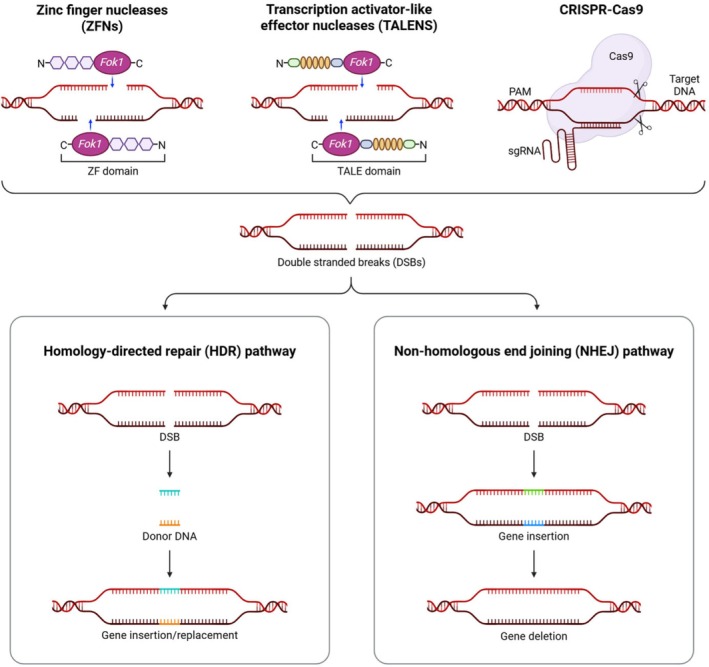
Scheme showing the common molecular methods for gene modification. Here, zinc‐finger nucleases (ZFN), transcription activator‐like effector nucleases (TALEN), and the CRISPR/Cas9 system are shown, which induce double‐stranded breaks (DSB). Homology‐directed repair (HDR) or nonhomologous end joining (NHEJ) is used for gene insertion.

To overcome immune barriers, reduce virus transmission, and increase the compatibility of pig organs with the human immune system, scientists have used molecular tools for gene modification, such as zinc‐finger nucleases (ZFN), transcription activator‐like effector nucleases (TALEN), and the CRISPR/Cas9 system, to enable the accurate alteration of the pig genome (Figure 4).^16^ The advantages and limitations of these aforementioned methods are presented in Table 1.

**TABLE 1:** Summary and comparison of the advantages and limitations as well as the practical applications in xenotransplantation of three commonly used methods.

Feature	ZFNs	TALENs	CRISPR/Cas9 system
Description	An artificially designed endonuclease composed of a protein that recognizes a specific DNA sequence and a Fok1 cleavage domain, which induces DNA strand breaks	A precise genome‐editing tool developed by fusing a Fok1 nuclease domain to TALE repeats that bind DNA. DNA binding specificity is determined by repeat variable residues at positions 12 and 13 of each repeat. Two opposing TALENs bind nearby DNA sequences, and their Fok1 domains form DSBs	A gene‐editing tool derived from a natural bacterial defense mechanism against viruses
Advantages	Capable of inducing DNA strand breakage to activate repair processes, leading to modification of the target locus. Modern gene‐editing techniques, such as ZFNs, help overcome immunological barriers, reduce the risks of viral transmission, and enhance compatibility between animal organs and the human immune system	Modern gene‐editing techniques, such as TALENs, are capable of inducing DNA‐double‐stranded breaks, which can lead to gene silencing. These advancements help overcome immunological barriers, mitigate the risks of viral transmission, and enhance compatibility between animal organs and the human immune system	Characterized by high design flexibility, versatility, and easy retargeting due to straightforward cloning procedures and oligonucleotide synthesis; can introduce multiple genetic modifications simultaneously. Cytosine base editing provides a safer alternative to earlier genome‐editing tools by enabling C to T conversion without DSBs, effectively inactivating genes through premature stop codons; significantly accelerated the process of creating genetically modified pigs for xenotransplantation
Limitations	Requires advanced protein engineering, making its design and implementation challenging. The FokI cleavage domain is derived from bacteria, which may increase the risk of an immune response, despite likely low immunogenicity	Requires complex molecular cloning techniques and the assembly of repetitive sequences. Immunogenicity remains unknown, as the protein used in this technology is derived from *Xanthomonas* bacteria	The immunogenicity of the system depends on the origin of the Cas9 protein, which is typically derived from bacteria (e.g., *Streptococcus pyogenes*) and may trigger an immune reaction
Practical applications of xenotransplantation	Can be used for the k.o. of the *GGTA1* gene, which encodes the enzyme responsible for producing the Gal (α‐Gal) antigen, a primary target of the human immune system in xenotransplantation	Used as a molecular tool for gene modification to overcome immune barriers, reduce virus transmission, and increase compatibility of pig organs with the human immune system	Successfully applied to inactivate multiple xenoantigen genes in pigs, including *GGTA1*, *CMAH*, and *B4GALNT2*, in a single editing process; used as a molecular tool for gene modification to overcome immune barriers, reduce virus transmission, and increase compatibility of pig organs with the human immune system

*Note*: Here ZFNs, TALENs, and the CRISPR/Cas9 system are compared.

Abbreviations: DSB, double‐stranded break; k.o., knockout; TALEN, transcription activator‐like effector nuclease; ZFN, zinc‐finger nuclease.

ZFN is an artificially designed endonuclease consisting of two components: a protein designed to recognize a specific DNA sequence and the Fok1 cleavage domain, which induces DNA strand breakage. This damage activates repair processes in the cell, leading to the modification of the target locus.^39^ DNA strand breaks can be repaired by nonhomologous end joining (NHEJ), resulting in small insertions or deletions that alter the reading frame and potentially lead to gene k.o. The delivery of a homologous template DNA fragment enables sequence modifications. The aforementioned process is known as homology‐directed repair (HDR).^40^ An example of the practical application of ZFN is the *GGTA1* gene k.o., which encodes the enzyme responsible for producing the Gal (α‐Gal) antigen, the primary target of the human immune system in the context of xenotransplantation.^16^ The ZFN method requires advanced protein engineering, making it challenging to design and implement. Its immunogenicity is likely low; however, the FokI cleavage domain is derived from bacteria, which may increase the risk of an immune response.^20^

TALEN is another tool for precise genome editing, referring to the fusion of a Fok1 nuclease domain to so‐called TALE repeats, which bind to DNA.^41^ The specificity of DNA binding is determined by the so‐called repeat variable diresidues located at positions 12 and 13 of each repeat.^42^ Two opposing TALENs bind to DNA sequences close to each other, and the Fok1 domains lead to DNA‐double‐stranded (DDS) breaks. The cell repairs them mainly by NHEJ, which, as in the case of ZFN, can lead to gene silencing.^16^ This method requires complex molecular cloning techniques and the assembly of repetitive sequences. Its immunogenicity remains unknown, as the protein used in this technology is derived from *Xanthomonas* bacteria.^20^

CRISPR/Cas9 is a gene‐editing tool derived from a natural bacterial defense mechanism against viruses. This system has significantly accelerated the process of producing genetically modified pigs for xenotransplantation. The CRISPR/Cas9 system operates by introducing breaks in the DNA strand, which activate the cell's repair mechanisms. As a result, insertions, deletions, or precise integration of the delivered genetic material can occur. Cas9, functioning as molecular scissors, plays a key role in the aforementioned technique. It contains two domains: one, called HNH, is responsible for cutting one DNA strand, and the other, RuvC, cuts the other strand.^43^

Subsequently, repair processes are initiated, including NHEJ and HDR.^44^ After DNA cleavage, these intracellular repair pathways are activated. HDR enables the introduction of new DNA sequences, whereas NHEJ results in minor mutations at the cleavage site, leading to the inactivation of the targeted gene. Additionally, NHEJ is divided into two pathways: classical NHEJ and alternative NHEJ, which differ in their mechanisms and repair efficiency. The alternative pathway frequently leads to large deletions (Figure 4).^45^

HDR requires the presence of specific enzymes that enable the precise joining of DNA fragments. A crucial role is played by the RAD51 protein, which is responsible for recognizing homologous sequences and initiating strand exchange at matching sites.^46^

CRISPR/Cas9 has emerged as the most commonly used genome‐editing platform due to its simplicity, versatility, and ability to introduce multiple genetic modifications simultaneously. For instance, it has been successfully applied to inactivate multiple xenoantigen genes in pigs, including *GGTA1*, *CMAH*, and *B4GALNT2*, in a single editing process.^20^, ^47^ More recently, cytosine base editing has been developed as an alternative approach that enables the conversion of cytosine (Cyt) to thymine (Thy) without causing DDS breaks. This technique can effectively inactivate genes by introducing premature stop codons and is considered a safer alternative to earlier genome‐editing tools, such as ZFNs and traditional CRISPR/Cas9 systems.^48^, ^49^

Besides genome editing, new approaches are being developed to enhance the spatial and temporal control of gene expression. One such approach involves the use of inducible promoters, which allow researchers to control transgene activation in specific tissues or at particular developmental stages. These promoters can be triggered by compounds such as tetracycline or doxycycline, administered either during gestation or postnatally, enabling precise regulation of gene activity throughout the animal's life or after organ transplantation.^50^, ^51^ This method is particularly valuable for genes that may cause adverse effects if expressed ubiquitously, including those with functions limited to endothelial cells^52^ or pancreatic tissues.^53^

RNA interference offers an alternative gene‐silencing approach, particularly useful when complete gene k.o. could cause lethality. This technique utilizes small interfering RNAs (siRNAs) to selectively downregulate specific genes. For example, siRNAs have been effectively used to suppress the activity of porcine endogenous retroviruses (PERV)^54^, ^55^ and tissue factor,^56^ achieving gene‐silencing efficiencies exceeding 95%.

The CRISPR/Cas9 system is characterized by high design flexibility and easy retargeting, due to the use of straightforward cloning procedures and oligonucleotide synthesis. The immunogenicity of the system depends on the origin of the Cas9 protein, which is typically derived from bacteria such as *Streptococcus pyogenes*, and may trigger an immune reaction.^20^

#### 2.4.2 From exogenesis to chimeras

One step forward in xenotransplantation has been exogenesis, in which the aim is to increasingly humanize the animal heart structures by introducing human stem cell populations to substitute specialized tissues in the organ.^57^, ^58^ Exogenesis is a novel and promising strategy in xenotransplantation that aims to grow human‐like organs within animals, particularly pigs. This is achieved using a technique called blastocyst complementation, where specific genes in pig embryos (e.g., ETS variant transcription factor 2) are knocked out to prevent the development of certain tissues, including endothelium, which is the primary trigger causing tissue rejection. Then, human pluripotent stem cells are introduced to fill those gaps and form human tissues within the animal embryo.^59^ Recent studies have shown that by modifying human cells, such as adding genes like *BCL2* to enhance survival, it is possible to produce pig embryos with partially humanized organs, including vasculature.^60^ Although challenges remain, including differences in development speed and molecular communication between species, the latest research is exploring ways to enhance cell integration and function.^58^, ^59^ Overall, exogenesis could lead to safer and more compatible organs for human transplantation compared to traditional xenografts.^58^

The most recent studies focus on investigating the production of human–animal chimeras, especially human–pig chimeras, using interspecies blastocyst complementation as a possible route to grow transplantable human organs within pigs. In this method, pig embryos are genetically modified to lack specific genes that regulate organ development, creating developmental niches that human‐induced pluripotent stem cells (hiPSCs) can occupy and differentiate into target tissues, such as blood vessels or kidneys.^61^, ^62^ Previous studies, using rodents, achieved lower levels of human cell integration, demonstrating the major technical challenge posed by interspecies incompatibilities in development.^63^ Thus far, most studies aiming to produce interspecies chimeras using hiPSCs have been carried out in mouse models, but these experiments have exhibited limited efficiency.^64^, ^65^, ^66^ Later, Wu et al.^63^ demonstrated that hiPSCs were engrafted in both porcine and bovine preimplantation blastocysts. The hiPSCs are often cultured under optimized conditions, and the injection of hiPSCs into these niches has recently enabled the formation of humanized mesonephric tissue in pig fetuses, with up to 40%–60% human cell contribution in some organs by embryonic day 28.^67^ Overcoming barriers such as apoptosis, cell competition, ligand–receptor mismatches, and differences in developmental timing remains critical to increase the efficiency and safety of chimera formation, especially to avoid off‐target contributions, such as to the brain or germline.^58^ Although ethical considerations remain significant, these advances represent a crucial step toward producing patient‐specific human organs through chimeric strategies in pigs.^62^

### 2.5 Clinical applications

Xenotransplantation utilizes both whole organs and animal‐derived cells and tissues for human therapies. Key applications include the xenotransplantation of neural cells for the treatment of Parkinson's disease,^68^ pancreatic islet cell transplants for type 1 diabetes,^69^ and porcine hepatocytes for temporary liver support in patients with liver failure.^70^ Beyond cellular transplants, animal‐derived tissues such as heart valves^71^ and corneas^72^ serve as important supportive implants. These transplantation approaches aim to either temporarily or permanently restore organ function, depending on disease severity and technological capabilities.

#### 2.5.1 Neural cell transplantation

Neurodegenerative diseases such as Parkinson's and Huntington's diseases are increasingly prevalent and have limited treatment options. Cell replacement therapies have shown promise, particularly in the treatment of Parkinson's disease, using human fetal tissue; however, ethical concerns and limited availability restrict their broader application. This has prompted interest in alternative sources, such as porcine neuroblasts, which are abundant and share key characteristics with human neurons.^73^, ^74^

A notable example involves the xenotransplantation of fetal porcine neural cells into the caudate‐putamen region of the human brain for the treatment of Parkinson's disease. Research demonstrated a 7‐month graft survival with evidence of functional porcine dopaminergic neurons and glial cells within the host brain. Importantly, the transplanted porcine neurons successfully prolonged axons from the graft site into the recipient's brain tissue.^68^

Other phase I trials provided evidence that fetal porcine neural cells can be safely transplanted into patients suffering from Parkinson's and Huntington's diseases. In Parkinson's disease, unilateral ventral mesencephalic grafts resulted in a 19% motor improvement after 12 months, with some patients experiencing an improvement of over 30%. Graft survival was confirmed at 7 months, consistent with earlier findings. In Huntington's disease, striatal grafts were well tolerated, though without functional benefit. Importantly, there were no serious adverse effects and no evidence of porcine pathogen transmission or activation of PERVs in either case.^75^

Recent advances include the transplantation of genetically modified porcine neural precursors in immunosuppressed primates, demonstrating long‐term graft survival exceeding 18 months and associated clinical improvements.^74^ However, challenges remain regarding the optimal cell types, immune responses, and strategies to enhance graft integration and function, which require further investigation before broader clinical application.

#### 2.5.2 Pancreatic islet transplantation

Pancreatic islet transplantation represents another significant application, particularly for people with type 1 diabetes who must meet strict criteria, including a minimum 5‐year diabetes diagnosis. In this approach, porcine pancreatic islets are encapsulated and laparoscopically implanted into the peritoneal cavity. These transplanted islets begin producing insulin, helping regulate blood glucose levels while reducing dependence on external insulin administration. Crucially, studies indicate that encapsulated porcine islet transplantation can achieve therapeutic effects without requiring immunosuppressive drugs.^69^ More recent clinical follow‐up has shown sustained glycemic improvements and reduced insulin requirements in human patients up to 10 years *post transplantationem*, with no serious adverse events or cases of cancer reported. Most patients reported improved diabetes management and expressed a willingness to recommend the treatment or receive additional transplants.^76^

#### 2.5.3 Hepatocyte transplantation

Extensive research has also focused on hepatocytes for patients with liver failure. The cells were encapsulated and intraperitoneally implanted into baboons with liver failure. These capsules protect the hepatocytes from the recipient's immune response while allowing oxygen and nutrients to reach the cells. As a result, three of four baboons recovered. Although human trials have not yet been conducted, these findings confirm the method's effectiveness, paving the way for potential human therapies.^70^

#### 2.5.4 Porcine cornea transplantation

Another promising avenue is the use of porcine corneas, which are being investigated as a potential alternative to human corneal transplants due to their similar anatomical, biomechanical, and physiological properties. However, a major challenge lies in the surgical precision required for transplantation. Proper suturing of the donor and recipient corneal edges is crucial to ensure optimal wound approximation and therapeutic outcomes. Additionally, the thickness of the porcine cornea must closely match that of the human cornea, which is complicated by variations depending on the pig's age and breed, making donor selection difficult. Although the cornea is avascular, rejection can still occur. Genetically modified pig corneas elicit a weaker immune response than wild‐type corneas. Furthermore, decellularization techniques reduce the expression of Gal and NeuGc antigens and decrease antibody binding to porcine corneal tissue. Studies in monkeys show no immediate rejection, but an immune response still develops.^77^ Nonetheless, xenogeneic rejection remains a significant barrier. Recent studies in NHP revealed that complement activation and leukocyte‐mediated inflammation are key mechanisms of graft failure, even under immunosuppression.^78^ Despite its immune‐privileged status, the cornea is not immune to xenogeneic rejection. An in‐depth knowledge of immune tolerance pathways informs new strategies to promote long‐term graft survival.^79^ Particularly, long‐term studies in NHP have shown that systemic immunosuppression allows stable graft survival without severe adverse effects, providing a strong foundation for the clinical application of porcine corneal xenotransplantation.^80^

#### 2.5.5 Biological heart valves

Biological heart valves of animal origin have long been used in cardiac surgery and hold significant importance in xenotransplantation. To make animal valves safe for human use, tissues can be decellularized. The aim of these processes is to remove donor cells and preserve the extracellular matrix, thereby reducing the risk of immune rejection after implantation.^81^, ^82^

The preference for using porcine or bovine valves has been a subject of controversy. Valves derived from bovine pericardium tend to have higher immunogenicity and often require more intensive preparatory procedures.^83^ Clinical data indicate that patients receiving porcine valves more frequently require reoperation compared to those with bovine valves. Nevertheless, porcine valve implantation is associated with better long‐term survival.^84^ However, other studies show that bovine pericardial valves often offer better late survival and lower pressure gradients compared to porcine prostheses in aortic valve replacement, although structural deterioration rates may be similar.^85^ The tissue is treated alongside anticalcification techniques to reduce antigenicity while maintaining durability of ~15–20 years.^86^ Recent research efforts continue to refine processing methods and pharmacological treatments to enhance the longevity and biocompatibility of bovine‐based bioprosthetic valves.

#### 2.5.6 Organ transplantation

##### Heart transplantation

In 2023, a 58‐year‐old man with heart failure received a genetically modified pig heart containing 10 edited genes. Initially, the transplant functioned correctly, but progressive diastolic heart failure developed over time, accompanied by ventricular wall thickening and near‐total loss of contractile function. Due to these complications, the patient transitioned to comfort care after 40 days. Histological analysis revealed capillary endothelial damage, interstitial edema, and an early onset of fibrosis.^87^

Recent anatomical and physiological studies highlight critical differences between pig and human hearts that may influence xenotransplantation outcomes. Variations in coronary artery structure and myocardial metabolism could contribute to early graft dysfunction, such as diastolic failure and fibrosis observed clinically. Consequently, gene‐editing strategies are now also targeting the improvement of pig heart compatibility with human physiology. Advances in immunosuppression and perioperative care, alongside approaches to protect endothelial cells and microvasculature, are crucial for enhancing graft survival and function.^88^

##### Kidney transplantation

According to the NYU Langone Health report (2025), the transplantation of a kidney from a genetically modified pig represents a breakthrough in xenotransplantation. The procedure was performed in 2024 on a 53‐year‐old female patient with kidney failure. The patient was under strict clinical observation; 130 days postprocedure, the decision was made to remove the transplanted kidney due to deterioration of health that coincided with the reduction in immunosuppressive therapy, necessary to combat an infection not directly related to the organ. The patient returned to dialysis.^89^

##### Liver transplantation

The liver presents particular challenges in xenotransplantation, as it faces not only traditional immunological barriers but also the risks of thrombocytopenia and coagulopathy.^90^ In 2025, a heterotopic liver transplant was performed using a pig liver with six genetic modifications. The recipient was a brain‐dead patient. Over a 10‐day period, organ function was monitored, and the results showed that the porcine liver was capable of producing bile, albumin, and liver enzymes. Vascular flow was normal, and no thrombocytopenia was observed. The transplant remained functional until the end of the study.^91^

##### Lung transplantation

Currently, intensive preclinical research is being conducted on lung xenotransplantation, such as the recently reported pig‐to‐human lung transplantation into a brain‐dead recipient.^92^ Despite the removal of the galactosyltransferase gene (*GalTKO*), transplanted lungs continue to be rejected by the baboon immune system. To improve transplant function, additional genetic modifications were implemented, involving the introduction of human genes responsible for regulating the complement system, coagulation cascade factors, anti‐inflammatory enzymes, and self‐recognition receptors. Furthermore, k.o. of the β4Gal xenoantigen was performed. These modifications were tested in various combinations. In most cases, lungs functioned briefly, typically up to 24 h, due to inflammation and loss of vascular integrity in the transplanted lung. In one case, through the application of advanced genetic modifications along with additional pharmacological blockade of numerous pro‐inflammatory immune mechanisms, both innate and adaptive, the recipient survived 31 days. Although these results represent an important advancement in the clinical application of lung xenotransplantation, further research is necessary.^14^

## 3 CONCLUSIONS

Transgenic animals represent a promising avenue for advancing xenotransplantation. The growing demand for transplantable organs has motivated the scientific community to seek innovative solutions, including breeding genetically modified pigs as potential organ donors, as transplants from wild‐type pigs trigger severe immune rejection. Modern gene‐editing techniques, such as CRISPR/Cas9, ZFNs, and TALENs, help overcome immunological barriers, reduce risks of viral transmission, and enhance compatibility between porcine organs and the human immune system. Despite impressive progress, xenotransplantation remains experimental and requires further research. It is important to remember that even as potential donors, animals are sentient beings deserving humane treatment and dignified living conditions. All xenotransplantation development strategies must consider clinical efficacy while prioritizing ethical principles and animal welfare. Future prospects include alternative approaches, such as interspecies chimera development and organ bioengineering through three‐dimensional bioprinting. However, key challenges remain: ensuring effective vascularization of structures, developing bioinks that best mimic the properties of natural tissues, and scaling processes to the level of complex organs containing multiple cell types. Interspecies blastocyst supplementation, supported by precise gene‐editing techniques such as CRISPR/Cas9, is a promising strategy for addressing the organ shortage problem in transplantation. This method involves genetically disabling the development of a specific organ in the host embryo, which creates a developmental niche for donor cells. Embryonic stem cells or induced PSCs are then introduced into the blastocyst, which controls the development of the missing organ. The resulting organ mainly consists of donor cells and can be used for transplantation.

## AUTHOR CONTRIBUTIONS

**Julia Motławska:** Conceptualization; writing – original draft. **Ana Amaral:** Writing – review and editing. **Marta Cerveira‐Pinto:** Writing – review and editing. **Paweł Kordowitzki:** Visualization; writing – original draft; writing – review and editing.

## FUNDING INFORMATION

Paweł Kordowitzki received the IDUB Mobility Grant of the Nicolaus Copernicus University.

## CONFLICT OF INTEREST STATEMENT

None to declare.

## ETHICS APPROVAL STATEMENT

Not applicable.

## CONSENT FOR PUBLICATION

All authors approved the submission of this article.

## Data Availability

Not applicable.
